# Supplementary material for: A qualitative review of challenges in recruitment and retention in obstetrics and gynecology in Ireland: The consultants’ solution based perspective
Source: PLoS One. 2022 Dec 28;17(12):e0279635. doi: 10.1371/journal.pone.0279635 (PMC9797065; doi:10.1371/journal.pone.0279635)
Supplement: S1 File — (DOCX) [file pone.0279635.s001.docx]

**Interview Schedule for Qualitative Study Seeking Consultant’s Insights and Solutions to Current Challenges**

1. Could you outline your background in obstetrics and gynaecology?
2. How do you see the issue of recruitment and retention of trainees to the specialty?
3. How do you think obstetrics and gynaecology has changed as a career option during your time in the specialty?
4. What is your perception of morale amongst trainee and consultant doctors working in obstetrics and gynaecology at present?
5. What is your impression of the media impact on morale of trainees and consultants working in the specialty?
6. What is your impression of the impact of the medico-legal climate on trainees and consultants working in the specialty?
7. What other factors do you think are affecting morale?
8. What is your perception of how morale is influencing recruitment and retention to the specialty?
9. Are there other factors that might contribute to problems with recruitment and retention?
10. What solutions might you propose to improve recruitment and retention in obstetrics and gynaecology?
11. Is there anything I have not asked you or that you would like to add?
